# Multivariate Modeling of Proteins Related to Trapezius Myalgia, a Comparative Study of Female Cleaners with or without Pain

**DOI:** 10.1371/journal.pone.0073285

**Published:** 2013-09-04

**Authors:** Jenny Hadrevi, Bijar Ghafouri, Britt Larsson, Björn Gerdle, Fredrik Hellström

**Affiliations:** 1 Department of Integrative Medical Biology, Anatomy, Umeå University, Umeå, Sweden; 2 Centre for Musculoskeletal Research, Department of Occupational and Public Health Sciences, Faculty of Health and Occupational Studies, University of Gävle, Umeå, Sweden; 3 Rehabilitation Medicine, Department of Medicine and Health Sciences (IMH), Faculty of Health Sciences, Linköping University and Pain and Rehabilitation Centre, County Council of Östergötland, Linköping, Sweden; 4 Occupational and Environmental Medicine, Department of Clinical and Experimental Medicine, Faculty of Health Sciences, Linköping University and Centre of Occupational and Environmental Medicine, County Council of Östergötland, Linköping, Sweden; Universidad Europea de Madrid, Spain

## Abstract

The prevalence of chronic trapezius myalgia is high in women with high exposure to awkward working positions, repetitive movements and movements with high precision demands. The mechanisms behind chronic trapezius myalgia are not fully understood. The purpose of this study was to explore the differences in protein content between healthy and myalgic trapezius muscle using proteomics. Muscle biopsies from 12 female cleaners with work-related trapezius myalgia and 12 pain free female cleaners were obtained from the descending part of the trapezius. Proteins were separated with two-dimensional differential gel electrophoresis (2D-DIGE) and selected proteins were identified with mass spectrometry. In order to discriminate the two groups, quantified proteins were fitted to a multivariate analysis: partial least square discriminate analysis. The model separated 28 unique proteins which were related to glycolysis, the tricaboxylic acid cycle, to the contractile apparatus, the cytoskeleton and to acute response proteins. The results suggest altered metabolism, a higher abundance of proteins related to inflammation in myalgic cleaners compared to healthy, and a possible alteration of the contractile apparatus. This explorative proteomic screening of proteins related to chronic pain in the trapezius muscle provides new important aspects of the pathophysiology behind chronic trapezius myalgia.

## Introduction

Trapezius myalgia remains a major problem in work tasks with high exposure to awkward working positions, repetitive movements and movements with high precision demands [1]. Although several pathophysiological models have been suggested (*reviewed by Visser and Van Dieen 2006*) [2], the aetiology behind trapezius myalgia is insufficiently elucidated. Several studies have investigated trapezius muscle structure and biochemistry in both healthy subjects and in patients with chronic trapezius myalgia [3], [4]. In myalgic muscle, an increased fiber area of type 1 muscle fibers has been recognized [4], [5]. An inability to utilize oxygen in myalgic trapezius has been suggested based on changes in cyclooxygenase (COX) protein complex IV of the mitochondrial respiration chain. COX negative fibers have been found in muscle biopsies from women suffering from trapezius myalgia [6]. Other studies have shown COX negative fibers to be connected to work exposure, as COX negative fibers prevailed in both pain and pain free subjects with the same kind of work exposure [7]. However, there seems to be a substantial biological variation in the presence of COX-negative fibers among subjects with and without trapezius myalgia [7]. There are also reports of muscle alterations probably due to mitochondrial disturbances such as moth-eaten and ragged-red fibers [7]. In more recent studies, using microdialysis (MD) [8], increased concentrations of pyruvate and lactate in the myalgic trapezius muscle have been reported [9], [10], [11], suggesting alterations in energy metabolism. Furthermore, several studies using MD report increased interstitial levels of serotonin and glutamate in chronic trapezius myalgia [12]. The majority of biochemical studies conducted on myalgic trapezius muscle has addressed a few biochemical parameters in each study [1], [12]. A more complete picture of the underlying mechanisms behind trapezius myalgia has not yet been obtained; hence more comprehensive studies are needed.

Advances in research technology allow investigation of a substantial part of the protein content in a tissue. Two-dimensional difference gel electrophoresis (2D-DIGE) [13] in combination with mass spectrometry has been used for simultaneous screening of differentially expressed proteins in different chronic pain conditions. The proteome of the cerebrospinal fluid in healthy subjects, in patients with herniated disc and idiopathic back pain have been investigated [14], [15], [16]. Also, nerve samples from patients with complex regional pain syndrome (CRPS) have been analyzed in order to detect up/down regulated proteins [17]. Proteomics of serum have been used for the identification of possible biomarkers of chronic endometriosis [18], [19]. In addition, several proteomic studies have investigated human skeletal muscle exposed to high altitude [20], bed rest [21], [22], and exercise [23]. Also, differences in protein content comparing different human muscles has been studied [24], [25], showing protein patterns related to muscle function.

The aim was to explore differentially expressed proteins in muscle biopsies of the trapezius muscle in female cleaners with chronic trapezius myalgia compared to healthy female cleaners using a combination of a proteomic screening method and multivariate modeling.

This explorative comprehensive screening is expected to provide new clues regarding differences in protein content between myalgic and healthy trapezius muscle. The clues presented allows the creation of new hypotheses for the pathophysiology of myalgic muscle, which in the future will provide a better understanding regarding the maintenance of myalgia, and facilitate the creation of diagnostic tools and treatment. This screening provides an important hallmark as it presents a novel exploration of the myalgic muscle tissue proteome.

## Subjects and Methods

### Subjects

Twelve female cleaners with work-related trapezius myalgia (MYA) and twelve female cleaners without work-related trapezius myalgia (CON) participated in the study. The mean (± one standard deviation, ±1 SD) age, height and mass for MYA was 42±8 years, 165±6 cm, 71±12 kg and for CON 41±8 years, 165±6 cm and 68±16 kg. The main work duty of all cleaners was manual floor cleaning requiring a high work demand on the trapezius muscle [26]. Trapezius myalgia was considered work related when reported to have started while working as a cleaner and when it worsened during the working day. Pain duration for the participants in the myalgia group was on average 7 years. The female cleaners comprising the control group reported no neck pain at all or neck pain for a maximum of 2–3 days during the previous 12 months.

Subjects taking oral steroids or non-steroidal anti-inflammatory drugs were excluded from this study. No one suffered from trauma to the neck region, diabetes mellitus, rheumatic diseases, generalized pain, or fibromyalgia, hence fulfilling the criteria of the American College of Rheumatism 1990 [27]. After receiving written information about the study, all subjects signed a consent form that was in accordance with the Declaration of Helsinki. The study was granted ethical clearance by the ethics committees of the University of Lund and University of Linköping (dnr: Dm131-07).

### Clinical Examination

Participants underwent a standardised clinical examination [28] to ensure that the myalgic patients and healthy subjects met the inclusion criteria. The clinical examination included questions on pain, tiredness and stiffness on the day of examination. Also, physical tests were performed, including range of motion and tightness of muscles, pain threshold and sensitivity, muscle strength and palpation of tender points. The trapezius myalgia diagnosis included: neck pain, tightness of the trapezius muscle (a feeling of stiffness in the descending part of the trapezius muscle during lateral flexion of the head) and palpable tender parts in the trapezius muscle. The range of motion of the columna had to be normal or just slightly decreased [28]. The clinical examination protocol allows the examiner to exclude subjects with pain in the trapezius region likely referred from painful tendons or nerve compressions in the neck and shoulder area.

### Biopsy Collection

Open surgical samples biopsies were taken from the descending part of the trapezius muscle. In the MYA group samples were taken on the myalgic side and in the CON group samples were obtained from the dominant side. If the subjects in the MYA group had bilateral trapezius myalgia the most painful side was used for biopsy collection. The incision was placed 2 cm lateral of the midpoint between the 7th process of the cervical spine and the lateral part of the acromion process. The skin and the subcutaneous area were infiltrated with 3–5 ml 5% xylocaine. Care was taken not to infiltrate the fascia or the muscle with xylocaine. A piece of muscle tissue, approximately 0.5 cm×0.5 cm×0.5 cm, was carefully removed and placed in a humid chamber for 15 min during transportation. The samples were then oriented and mounted for transverse sectioning in Optimal Cutting Temperature (OTC) compound (Tissue Tek, Miles laboratories, Naperville, Ill., USA) and frozen in chilled liquid propane and stored at −80°C until use.

### Two-Dimensional Difference Gel Electrophoresis (2-D DIGE) and Image Analysis

Unless otherwise stated, all chemicals were from GE healthcare, Uppsala, Sweden and of proteomic grade quality. The protocol has previously been evaluated [29]. The frozen muscle samples were suspended in lysis buffer (9.5 M Urea, 4% (v/w) CHAPS and 30 mM Tris Base) and homogenized with a Grinding Kit. Quantification of protein content was made with a 2D-Quant Kit. Labelling of protein samples, containing 50 µg of protein, was made with CyDye minimal dyes, Cy2, Cy3 and Cy5, in accordance to manufacturers’ protocol. The internal standard method was used [30] where a pooled internal standard containing sample from each of all the biopsy samples included in the analysis, labelled with Cy2 was incorporated. Twelve biopsies from twelve different patients suffering from trapezius myalgia and twelve biopsies from twelve different healthy subjects were alternately labelled with Cy5 and Cy3. Added to each gel three samples, Cy2, Cy3 and Cy5, from healthy, myalgic and pooled internal standard were pooled to be simultaneously separated. An equal volume of lysis buffer was added. An IPG-buffer pH 3–11 was added to reach a concentration of 2% (w/v). DeSteak™ rehydration solution was added to a final volume of 450 µl. The samples were applied onto 24 cm 3–11 Non-Linear (NL) Immobilised pH gradient (IPG) strips and rehydrated at room temperature in the dark for 16 hours. The first dimension was run using Amersham Ettan™ IPGphor unit applying 300 V for 900 Vhrs, 600 V for 1800 Vhrs, 1000 V for 3000 Vhrs, 5000 V for 55000 Vhrs. Prior to the second dimension the gel strips was equilibrated for 10 minutes in equilibration buffer (50 mM 1.5 M Tris HCl pH 8.8, 6 M Urea, 30% (v/v) Glycerol (87%), 2% (w/v) SDS, trace of Bromophenol Blue and 0.5% DTT). Gels were then alkylated by further equilibration for 10 minutes in the same buffer, containing 4.5% (w/v) Iodoacetamide instead of DTT. The second dimension was conducted by using the Ettan DALT six apparatus as stips were loaded onto a 12.5% acrylamide gel. Gels were run simultaneously at 5 W per gel, 600 V, 400 mA for 30 minutes, followed by additional 5 hours or until the blue front reached the bottom of the gel, with 17 W per gel 600 V 400 mA of a constant temperature of 15°C. Gels were then immediately scanned with a Typhoon™ 9410 scanner, using 488 nm laser and emission filter of 520 BP40 for Cy2 labelled proteins, 532 nm laser and emission filter 580 nm BP30 for Cy3 and 633 nm laser and 670 nm BP30 for Cy5. The scanned image was further processed using ImageQuant™ V5.2, before protein abundance was determined and statistical evaluation was made using DeCyder™ V6.5. After differential in gel analysis (DIA), gels were batch processed with exclusion filters set at max slope: 1.0, min spot volume 20.000. Protein spots that occurred in >50% of the gels were included in the analysis.

### Protein Identification by LC-MS/MS

In gels used for protein identification 450 µg unlabelled proteins were loaded and analysed as above. Protein spots were excised using Ettan™ Spot Handling Workstation, with a ∅ 2.0 mm picker head. The picked protein spots were digested with trypsin (Promega/SDS Biosciences, Falkenberg, Sweden). Briefly, the gel pieces were washed with a mixture of acetonitrile/ammonium bicarbonate, dehydrated with acetonitrile and incubated with 20 µl of 20 µg/ml trypsin overnight at 37°C. The supernatant was transferred to a new tube and the peptides further extracted from the gel by incubation in 50% acetonitrile/5% trifluoroacetic acid for about 3 hours at room temperature with occasional mixing. The supernatant obtained by the two steps pooled, dried by SpeedVac and dissolved in 6 µl of 0.1% formic acid. Peptides were analysed using an on-line nano-flow HPLC system (EASY-nLC; Proxeon, Bruker Daltonics) in conjugation with the mass spectrometer HCTultra PTM Discovery System (Bruker Daltonics). A 100 mm×75 µm C18 column was used for separation at a flow rate 300 nL/min. The gradient buffers used were 0.1% formic acid in water (buffer A) and 0.1% formic acid in acetonitrile (buffer B) and a linear gradient from 0–100% buffer B in 40 min was used for separation. The automated online tandem MS analysis was performed using collision induced dissociation of peptide ions.

### Statistical Analysis

Statistical evaluation of the protein abundance in the 2D-DIGE analysis was made using the Biological Variation Analysis (BVA) module in DeCyder™ V6.5 and multivariate modeling was performed using SIMCA-P version 12 (Umetrics AB, Umeå, Sweden). In all statistical analyses, the log of the standardized abundance derived from the ratio of each pooled protein group (each gel) normalized by the internal standard was used. In the Differential In-gel Analysis (DIA) 1330 protein spots were included in the BVA. Spots with slope values <1.0 were considered non-protein spots and not included in the analysis. In data-sets from 2D-DIGE analysis the number of variables (protein spots) greatly exceeds the number of observation and the variables are often highly correlated.

Multivariate projection methods have the advantage in being able to deal with such data. In this study both principal component analysis (PCA) and projections to latent structures with discriminant analysis (PLS-DA) was used. PCA is an unsupervised projection method to extract and display systematic variation between×variables. PLS-DA is a supervised linear regression method to find a relationship between×variables and in this case a binary Y vector using class membership data. This method, in contrast to classical statistical methods, does not assume that a high subject-to-variables ratio is present (5–10). Such requirements are not required for PCA or PLS, in fact PLS-DA can handle ratios lower than 1.0. In the multivariate data analysis (PCA and PLS-DA) the spot volume ratios were mean centered and scaled for unified variance. PCA was used to detect multivariate outliers among the observations - e.g., due to technical problems with gels - and as a general overview of the data. In the PLS-DA modeling the number of PLS-DA components was determined by full cross-validation (SIMCA-P version 12, Umetrics AB, Umeå, Sweden). This method keeps part of the data out from the model development to assess the predictive power of the model and was used to test the significance of the components. Hence, this validation technique increases the stability of the results. Such validation is not implemented in other common statistical packages e.g., SPSS. The PLS-DA model was validated by both response permutation tests and CV-ANOVA on the residuals [31]. Only protein spots present in at least 50% of the gels and with ratio of between subject variance vs. within subject variance falling within the 15^th^ percentile of an F-distribution (equal to using a cut-off at p-value = 0.3 in a one-way ANOVA) were selected for PLS-DA modeling and identification.

### Database Searches

Spectra were processed by Bruker Daltonics DataAnalysis 3.4 (Bruker Daltonics, Bremen, Germany) and resulting MS/MS data were searched in NCBInr and Swiss-Prot database on MASCOT server (www.matrixscience.com). Database search parameters were set as follows: the enzyme trypsin was used; up to one missed cleavage was allowed; fixed modification included were carbamidomethylation of cysteine and oxidation of methionine; mass tolerance for MS precursor ion was 0.8 Da and for MS/MS fragment ion was 0.6 Da; and charge states were varied. Criteria for identification of a protein were at least 3 peptides of the protein should be identified with a MASCOT score over 25 and an expectation value <1.

## Results

**Figure 1:**
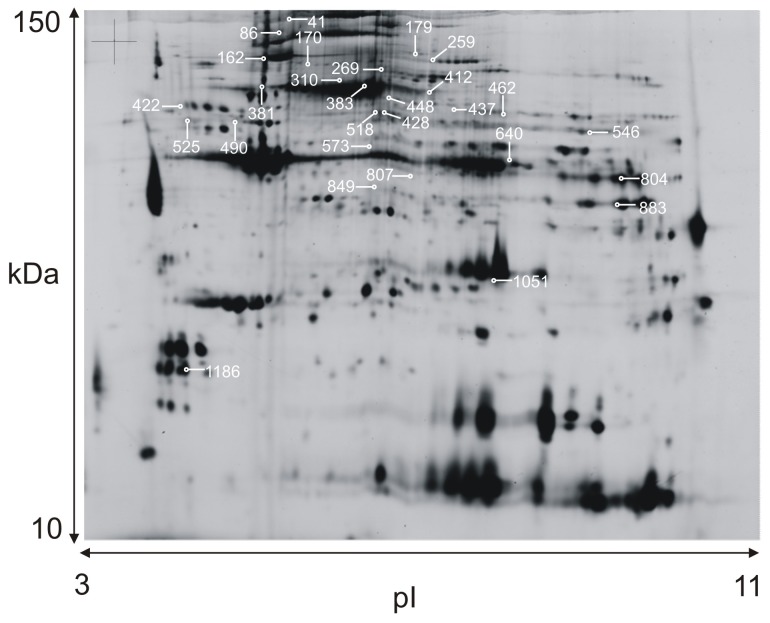
A typical 2-DE gel pattern of human trapezius muscle homogenate. (pH 3–11 and 12.5% SDS). Identified proteins marked with spot ID numbers, available in table 1.

A typical 2-DE protein pattern of human trapezius muscle is shown in **figure 1**. Altogether 1330 protein spots were detected. Eight-hundred forty-seven protein spots that were presented in at least 50% of the gels were included in the analysis of protein spot intensity in biopsy samples taken from controls and myalgic subjects. Of these, 170 protein spots met the criteria for identification, generating 162 identified proteins **(table S1)**.

**Figure 2:**
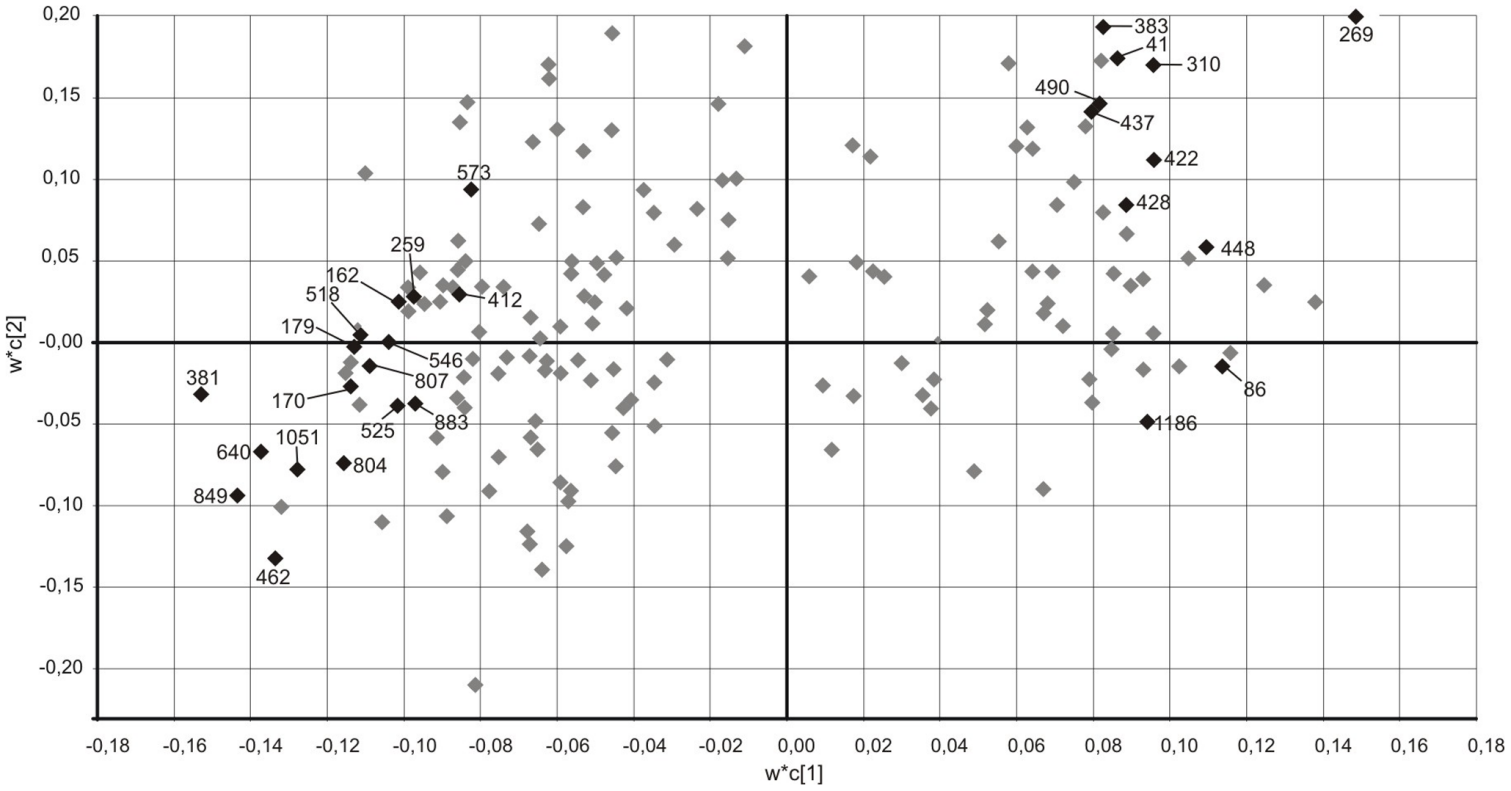
PLS-DA weight plot. Weight plot (w*c[1]/w*c[2]) from the PLS-DA model of healthy and myalgic muscle based on 170 protein spots. Situated on the left in the figure are proteins with a higher abundance in the myalgic muscle and on the right, proteins with a higher abundance in healthy muscle. Analyzed spots (◊) with variable of importance values (VIP)<1.

Of selected protein spots for identification 37 showed VIP-values of 1 or higher and 7 with a VIP-value of 1.5 or higher and yielding 28 unique proteins separating healthy and myalgic muscle (**figure 2**). Some proteins were identified in multiple isoforms. The 28 identified proteins were considered to belong to five groups based on their function: *metabolic (*n = 10), *contractile* (n = 8), *acute response* (n = 3), *structural* (n = 5), and *other* (n = 2) proteins (**table 1**).

**Table 1:** Protein Spot with Variable of importance (VIP) value >1.0 in the PLS-DA multivariate model.

ID (spotnr)	Protein ID (SwissProt)	Protein	*Mw*/*pI*	MS Score	No. of peptides	Av. Ratio (MYA/CON)	VIP PLS-DA
**Metabolic**							
269	P11217	Glycogen phosporylase, muscle form	97.5/6.6	134	4	1.47	1.9
462	P14618	Pyruvate kinase isoenzymes M1/M2	58.5/8.0	472	14	−1.16	1.7
640	P13929	Beta Enolase	46.8/7.7	1010	16	−1.11	1.6
804	P04075	Fructose bisphosphate adolase A&C	39.4/8.3	2795	14	−1.18	1.4
170	Q99798	Aconitate hydratase, mitochondrial	86.1/7.4	146	4	−1.40	1.3
310	Q02218	2-oxoglutarate dehydrogenase, mitochondrial	117/6.4	282	7	1.23	1.3
518	P13645	Creatine kinase, M-type	43/6.8	142	2	−1.16	1.3
546	P25705	ATP synthase, subunit alpha	55/8.3	129	4	−1.46	1.2
883	P04406	Glyceraldehyde-3-phosphate dehydrogenase	35.9/8.6	2062	14	−1.16	1.2
437	P36871	Phosphoglucomutase-1	61.7/6.3	797	19	1.07	1.1
**Contractile**							
381	P60709	Actin, cytoplasmic 1	41.6/5.3	64	2	−1.24	1.8
41	P62736	Actin aortic smooth muscle	42.4/5.23	99	3	1.36	1.3
383	Q00872	Myosin binding protein C, slow type	129/5.8	273	10	1.18	1.3
448	P12883	Myosin-7, slow	223/5.6	207	6	1.25	1.3
1186	Q96A32	Myosin regulatory light chain 2, skeletal muscle isoform	19.01/4.9	93	13	1.83	1.2
162	P13533	Myosin-6, fast	223.7/5.6	140	8	−1.42	1.2
573	Q9UKX2	Myosin-2	224/5.6	638	23	−1.12	1.1
412	P12883	Myosin-7, slow	223/5.6	93	6	−1.52	1.0
**Acute response**							
86	P08107	Heat shock 70 kDa protein	70.2/5.5	944	25	1.16	1.4
1051	P07451	Carbonic anhydrase 3	29.4/6.9	146	13	−1.32	1.5
422	P01009	Alpha-1-antitrypsin	46.7/5.4	99	2	1.31	1.2
**Structural**							
849	P04264	Keratin, type II cytoskeletal 1	66/8.1	382	13	−1.28	1.7
259	O75112	LIM domain-binding protein 3	77/8.5	155	2	−1.40	1.2
525	P07437	Tubulin beta chain	50/4.8	787	16	−1.17	1.2
428	P40123	Adenylyl cyclase associated protein 2	53/5.9	135	4	1.20	1.1
490	Q8TD99	Desmin	53.5/5.2	1059	39	1.28	1.1
**Other**							
179	P02768	Serum albumin	66.5/5.7	381	13	−1.13	1.4
807	P82650	28S ribosomal protein S22, mitochondrial	41/7.7	130	4	−1.27	1.3

Proteins identified with LC-MS/MS, MALDI-TOF-MS/MS. ID is the same as in figure 2 (Weight-plot PLS-DA).

The *Metabolic proteins* identified and of interest for separating healthy trapezius from myalgic trapezius were mainly related to glycolysis. In the myalgic muscle there were a higher abundance of glycogen phosphorylase; muscle form, 2-oxoglutarate dehydrogenase; mitochondrial, and phosphoglucomutase-1. In healthy muscle there were a higher abundance of pyruvate kinase isoenzymes M1/M2, beta enolase, fructose bisphosphate adolase A&C, aconitate hydratase; mitochondrial, creatine kinase; M-type, ATP synthase; subunit alpha and glyceraldehyde-3-phosphate dehydrogenase. *Contractile* proteins that were more abundant in myalgic muscle were actin aortic smooth muscle, myosin binding protein C slow type, myosin-7 slow and myosin regulatory light chain 2 fast; skeletal muscle isoform. In healthy muscle there was a higher abundance of the contractile proteins: myosin-6 fast, myosin-2, myosin-7 slow and actin; cytoplasmic 1.

The identified *acute response* proteins that separated the two groups were heat shock 70 kDa protein and alpha-1-antitrypsin, more abundant in myalgic trapezius; and carbonic anhydrase 3 more abundant in healthy trapezius. *Structural proteins* found to be more abundant in myalgic trapezius were adenylyl cyclase associated protein 2 and desmin. In healthy trapezius muscle keratin; type II cytoskeletal 1, LIM domain-binding protein 3 and tubulin beta chain were more abundant.

Two *other* proteins of relevance for separation between healthy and myalgic trapezius were serum albumin and 28S ribosomal protein S22, mitochondrial, both more abundant in healthy trapezius muscle.

Through PLS-DA model validation using response permutation test (Q^2^ intercept = -0.05, Q^2^(cum) = 0.65) and CV-ANOVA of residuals (F = 2.94, p = 0.0023) a two component model with a total of 29% explained variance (R^2^(cum) = 0.29)(Component 1∶16%, Component 2∶13%) was used to avoid over fitting the data.

## Discussion

In this study, muscle biopsies from women with and without chronic trapezius myalgia and with the same external work exposure were compared using 2D-DIGE and PLS-DA. Proteins correlating to either myalgic or healthy subjects in the PLS-DA (**figure 2**) were grouped as metabolic, contractile, regulatory, structural, acute response and other proteins, based on their biochemical function (**table 1**).

### Acute Response Proteins

The expression levels of heat shock 70 kDa (HSP 70) protein and alpha-1-antitrypsin, proteins involved in stress and inflammatory responses, were increased in cleaners with chronic trapezius myalgia compared to healthy cleaners. HSP70 kDa protein is an ATP binding protein that stabilizes pre-existing proteins against aggregation, prevents mis-location and facilitates protein folding, [23], [32]. Its expression level is highly inducible and the synthesis is increased in response to multiple stressors e.g., hyperthermia [33], energy depletion [34], hypoxia [35] and reactive oxygen species [36]. Alpha-1-antitrypsin is an acute phase protein with a broad anti-inflammatory spectrum [37]. It is released by macrophages in response to inflammation and has been suggested to control inflammatory components associated with fibromyalgia in musculoskeletal connective tissue [38].

A decreased level of carbonic anhydrase III, involved in oxidative processes, was found in cleaners with chronic trapezius myalgia. Carbonic anhydrase III is expressed predominantly in skeletal muscle and has been suggested to be an indicator of muscle damage [39]. It has been proposed that this protein functions as an essential anti-oxidant agent in skeletal muscle [40]. Decreased levels of carbonic anhydrase III in the myalgic muscle may indicate an imbalance in the cellular redox potential in this condition.

Taken together, these facts might indicate activated inflammatory mechanisms and alterations in anti-oxidant protection in chronic trapezius myalgia. These results have to be confirmed in future studies.

### Alterations in Metabolic Proteins

**Figure 3:**
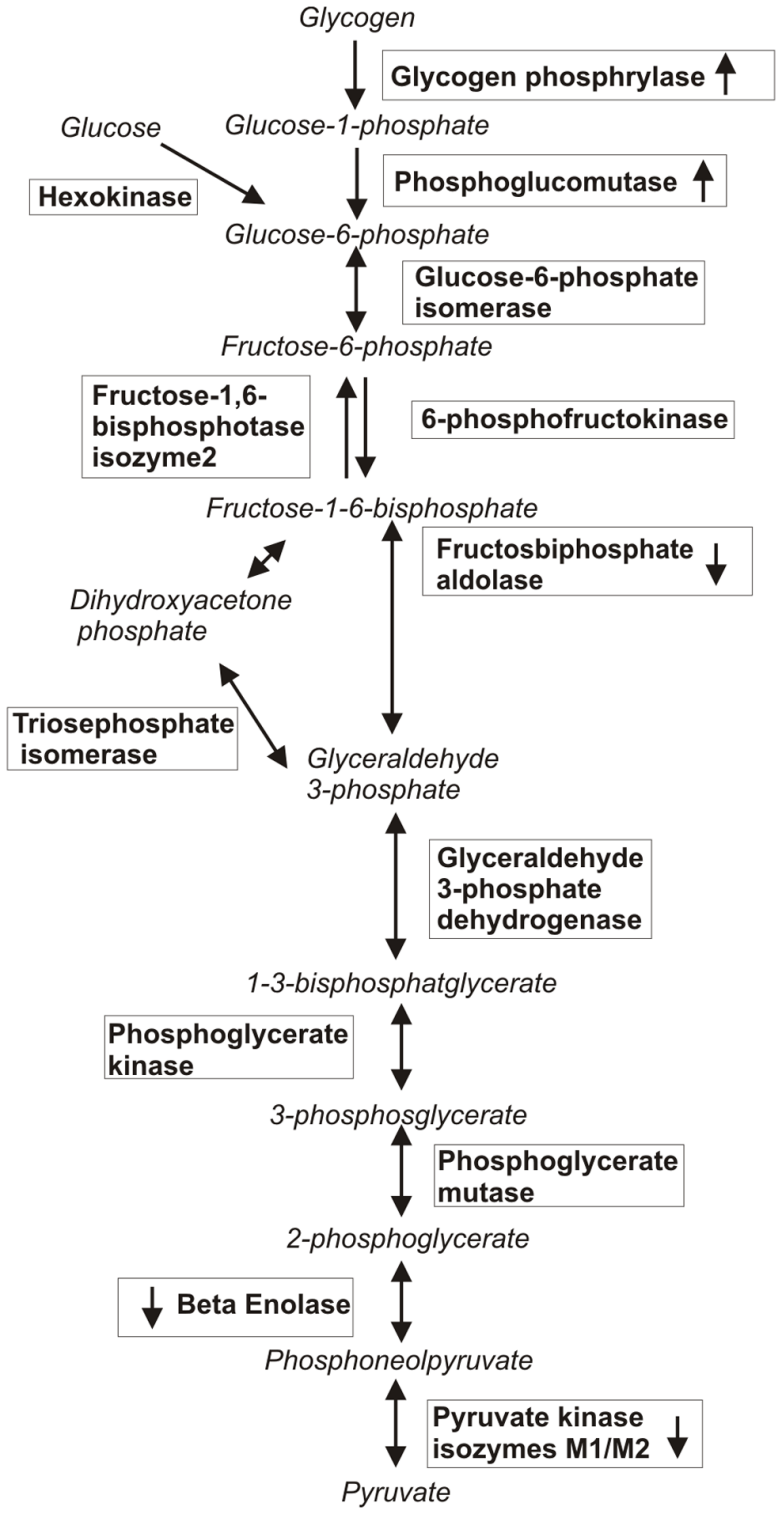
Schematic figure of the metabolic pathways: glycogenesis and glycolysis. Regulating enzymes are written in frames, arrows within frames indicate increased or decreased abundance in myalgic muscle compared to healthy muscle.

Glycogen phosphorylase and phosphoglucomutase-1 were increased in cleaners with trapezius myalgia (**table 1**). The catalytic action of glycogen phosphorylase is to break down muscle glycogen to Glucose-1-phosphate (G1P) and is the main regulatory step in glycogenesis (**figure 3**). Glycogen phosphorylase is activated by adrenaline and insulin through phosphorylation allosterically by adenosine monophosphate (AMP) or by substrate control through available inorganic phosphate. In healthy subjects glycogen phosphorylase transformation is related to energy state of the muscle cell [41]. The other major enzyme occurring with a higher abundance in myalgic muscle is phosphoglucomutase-1, which catalyzes the conversion of G1P to Glucose-6-phosphate which enters the glycolysis. The changes in the glycolytic enzymes of both glycogen phosphorylase and phosphoglucomutase-1 in skeletal muscle suggest an increased need or utilization of glucose from glycogen storages in the myalgic trapezius muscle in habitual daily activities. This is in contrast to skeletal muscles in healthy subjects where glycogen utilization at rest is low or absent [42]. Also, the energy utilization when the maximal oxygen consumption is below 35% relies mainly on plasma free fatty acid and glucose and not glycogen storages [43].

The other glycolytic enzymes that differed in abundance were fructose bisphosphate adolase A&C, beta enolase and pyruvate kinase isoenzymes M1/M2, all in the later part of the glycolysis (**figure 3**). The levels of abundance of these proteins were lower in MYA than in CON. Increased lactate concentrations have been shown to dissociate the key regulatory enzyme phosphofructokinase tetramers into dimers and thus reducing the enzymes activity and the glycolytic flux later part of the glycolysis [44]. Interstitial trapezius muscle concentrations of lactate have been reported to be significantly increased in chronic trapezius myalgia in six out of seven previous studies [45].

Pyruvate kinase isoenzyme M1/M2, showing a lower abundance in myalgic muscle compared to healthy, is a main glycolysis regulatory enzyme (**figure 3**) [46]. In muscle fibers, M1 is the major isoform [47]. In MD studies, increased pyruvate and lactate levels in myalgic trapezius muscle have been found [9], [10]. MD studies determine the extracellular concentrations of these substances while proteomics reflect both intra- and extracellular processes. One often proposed mechanism behind the increased interstitial concentrations of lactate is an increased reliance on anaerobic energy production in the myalgic muscle. However, increased concentrations of lactate can also occur during adequate oxygen provision [48], as lactate is also considered a systemically active metabolite capable of moving between cells and tissues, where it may be oxidized as a fuel or reconverted to form pyruvate or glucose [49], [50], [51]. Furthermore the interstitial concentrations of lactate is also dependent upon lactate dehydrogenase (LDH) and monocarboxylate transporters [52]. An increased anaerobic metabolism is not supported by the data presented in this study. It has been suggested that accumulation of pyruvate and lactate could occur if there is a higher flux through the glycolysis than the aerobic oxidative system can handle. Since none of the identified proteins related to the mitochondrial respiratory chain differed in abundance between cleaners with myalgic trapezius and healthy trapezius, this screening shows no evidence of pain related differences in the oxidative metabolism of female cleaners. Previous results regarding complex IV of the respiratory chain cyclooxygenase (COX) are in coherence with our results [6], [7].

### Alterations in Contractile and Structural Proteins

Comparative proteomic studies of different muscles, proteins related to oxidative metabolism have been shown to relate to the unique fiber type composition of the muscles [24], [25]. Previous results regarding fiber type composition of myalgic muscle in comparison to healthy are diverse [4], [6]. The main hypothesis supports an alteration towards more or larger type 1 fibers [5], [53]. Although there have been contradictory results both when comparing the fiber type content between myalgic and healthy muscle [54], and also when investigating differences in myosin heavy chain abundance [55]. The results of a higher abundance of type 1 fibers in myalgic muscle supports the theory that more type 1 fibers are recruited due to low load work [53]. As these results are not reproduced in other studies using larger research material [54], [56] this opens for other mechanisms explaining the muscle adaptation towards low-load work and muscle stiffness experienced by myalgic patients. Our results indicate a higher abundance of myosin light chain fast 2 regulatory in myalgic muscles. Myosin light chain fast 2 is predominant in fast contracting type 2 fibers [24], [25], [57], [58]. A higher occurrence of the protein in myalgic muscle might be due to an altered contractility of the myalgic muscle.

Creatine kinase M-type, was more abundant in the healthy muscle compared to the myalgic and is a metabolic protein more abundant in fast twitch fibers compared to slow twitch. Creatine kinase is used as a marker of energy turnover in the cell. Creatine kinase M produces phosphocreatine from mitochondrial ATP processes together with creatine. Phosphocreatine provides ATP for muscle contraction, or ion pumps, like the calcium pump for muscle relaxation and also serves as an energy buffer and an energy transporter [59].

Other contractile proteins that were more abundant in muscle biopsies from cleaners with trapezius myalgia were aortic smooth muscle actin, myosin binding protein C slow type and fragments of myosin 7 slow. Contractile proteins that were more abundant in healthy muscle were actin cytoplasmic 1 and fragments of myosin-6 fast, myosin-2 and myosin-7 slow. Proteins building the intermediate filaments and cytoskeleton are grouped as structural proteins. Healthy muscle has a higher abundance of keratin, LIM-binding protein 3 and tubulin beta chain. Myalgic muscle has a higher abundance of desmin and adenylyl cyclase associated protein 2. Desmin is a major intermediate filament of muscle fibers. The lack of desmin results in muscle dystrophy with disruption of muscle fiber integrity. Muscles from knock-out mice lacking desmin also become progressively stiffer and accumulate increased collagen in a degenerating process [60]. The increased abundance of desmin in the biopsies from myalgic may indicate remodeling of the cytoskeleton or a muscle regeneration process. In agreement with this an increased myogenic activity and increased myonuclear content have been reported when comparing myalgic and healthy muscle [61]. Moreover, a recent proteomic study of the interstitium of chronic trapezius myalgia indicated profound proteomic alterations in myalgia [62]. At the present it is unclear whether these alterations in contractile and structural proteins are primarily linked to nociceptive/inflammatory or metabolic processes or whether they may be secondary consequences of having a muscle in persistent pain e.g., deconditioning, altered activation patterns etc.

### Methodological Considerations

Two-dimensional gel electrophoresis is a useful comprehensive method, but choice of gel composition and buffer solutions will determine the type of proteins detected [63]. High molecular weight proteins are difficult to isolate on 2-DE and low molecular weight proteins are difficult to identify because only a few proteolytic fragments are generated by the tryptic digestion and so the database search leads to an uncertain identification. It is also important to keep in mind that what can be visualized by the staining method used in this study is only a top fraction of the proteins expressed in human trapezius muscle, and it can be expected that there are still several proteins at low abundance below the detection level that have not been evaluated.

### Conclusions

Proteomic analyses of biopsies from women with trapezius myalgia provide new insights into biological mechanisms that might reveal important aspects of the pathophysiology of trapezius myalgia. A variety of proteins that are involved in glycolysis, tricaboxylic acid cycle, contractile apparatus, cytoskeleton and acute response processes showed differential expression on the 2-DE gels. The results suggest altered metabolism, a higher abundance of proteins related to inflammation in myalgic cleaners compared to healthy, and a possible alteration of the contractile apparatus. Further studies will be required to provide a complete picture of the protein patterns of myalgic muscle. However, results presented here show extensive alterations in the proteome of myalgic muscles and provide new clues concerning the pathophysiological mechanisms behind chronic myalgia. These results are important in the creation of new hypotheses for the pathophysiology of myalgic muscle which in a future aspect could facilitate diagnosis and treatment of myalgic patients.

## Supporting Information

Table S1 **Identified proteins by LC-MS/MS or MALDI-TOF-MS/MS.**
(DOCX)
