# Supplementary material for: Multivariate Modeling of Proteins Related to Trapezius Myalgia, a Comparative Study of Female Cleaners with or without Pain
Source: PLoS One. 2013 Sep 4;8(9):e73285. doi: 10.1371/journal.pone.0073285 (PMC3762788; doi:10.1371/journal.pone.0073285)
Supplement: Table S1 — Identified proteins by LC-MS/MS or MALDI-TOF-MS/MS. (DOCX) [file pone.0073285.s001.docx]

Table S1.

| **Protein ID (SwissProt)** | **Protein** | **Mw/pI** | **No. of**  **peptides** | **MS**  **Score** |
| --- | --- | --- | --- | --- |
| P62736 | Actin aortic smooth muscle | 42.4/5.23 | 3 | 99 |
| P31948 | Stress –induced-phosphoprotein 1 | 62.64/6.40 | 4 | 45 |
| Q8NEC7 | Glutathione S-transferase, C terminal domain | 71/8 | 1 | 33 |
| Q13642 | Four and a half LIM domains protein 1 | 36.13/9.25 | 1 | 77 |
| P08107 | Heat shock 70 kDa protein 1A/1B | 70.2/5.5 | 3 | 70 |
| P08107 | Heat shock 70 kDa protein 1A/1B | 70.2/5.5 | 25 | 944 |
| Q00872 | Myosin-binding protein C | 129/5.8 | 27 | 960 |
| Q96NN9 | Apoptosis-inducing factor 3 | 67.4/9.2 | 1 | 35 |
| P68133 | Actin, alpha skeletal muscle | 41.82/5.23 | 13 | 540 |
| O75031 | Heat shock factor 2-binding protein | 37.6/5.4 | 1 | 37 |
| P02768 | Serum albumin | 66.47/5.67 | 13 | 275 |
| Q13642 | Four and a half LIM domains protein 1 | 36.13/9.25 | 3 | 109 |
| P02768 | Serum albumin | 66.47/5.67 | 25 | 674 |
| P11021 | 78kDa glucose regulated protein | 72.4/5.1 | 8 | 303 |
| P02768 | Serum albumin | 66.47/5.67 | 13 | 381 |
| P13533 | Myosin-6 | 223.7/5.6 | 8 | 140 |
| P51884 | Lumican | 36.66/6.17 | 3 | 140 |
| P02768 | Serum albumin | 66.47/5.67 | 9 | 231 |
| P02787 | Serotransferrin | 75/6.7 | 2 | 75 |
| O75112 | LIM domain-binding protein 3 | 77/8.5 | 2 | 155 |
| Q99798 | Aconitate hydratase, mitochondrial | 86.1/7.4 | 4 | 146 |
| Q99798 | Aconitate hydratase, mitochondrial | 86.1/7.4 | 19 | 752 |
| P68133 | Actin, alpha skeletal muscle | 41.82/5.23 | 8 | 218 |
| P68133 | Actin, alpha skeletal muscle | 41.82/5.23 | 7 | 264 |
| P68133 | Actin, alpha skeletal muscle | 41.82/5.23 | 6 | 209 |
| P51884 | Lumican | 36.66/6.17 | 2 | 126 |
| P62736 | Actin aortic smooth muscle | 42.4/5.23 | 4 | 102 |
| P68133 | Actin, alpha skeletal muscle | 41.82/5.23 | 9 | 325 |
| P11217 | Glycogen phosphorylase | 97.5/6.6 | 5 | 146 |
| P11217 | Glycogen phosphorylase | 97.5/6.6 | 4 | 162 |
| P11217 | Glycogen phosphorylase | 97.5/6.6 | 4 | 134 |
| P68133 | Actin, alpha skeletal muscle | 41.82/5.23 | 8 | 216 |
| P62736 | Actin aortic smooth muscle | 42.4/5.23 | 5 | 135 |
| P35609 | Alpha-actinin-2 | 103.8/5.31 | 17 | 757 |
| Q02218 | 2-oxoglutarate dehydrogenase | 117/6.4 | 7 | 282 |
| Q02218 | 2-oxoglutarate dehydrgenase | 117/6.4 | 2 | 96 |
| P02768 | Serum albumin | 66.47/5.67 | 9 | 326 |
| P04264 | Keratin, typ II cytoskeletal | 66/8.1 | 4 | 125 |
| P02768 | Serum albumin | 66.47/5.67 | 1 | 42 |
| Q00872 | Myosin-binding protein C | 129/5.8 | 10 | 273 |
| P02768 | Serum albumin | 66.47/5.67 | 10 | 221 |
| P60709 | Actin, cytoplasmic 1 | 41.6/5.3 | 2 | 64 |
| P02768 | Serum albumin | 66.47/5.67 | 7 | 142 |
| P68133 | Actin, alpha skeletal muscle | 41.82/5.23 | 9 | 231 |
| Q92679 | Myosin 7 | 223/5.6 | 12 | 429 |
| Q92679 | Myosin 7 | 223/5.6 | 6 | 93 |
| P02768 | Serum albumin | 66.47/5.67 | 31 | 1221 |
| Q92679 | Myosin 7 | 223/5.6 | 7 | 366 |
| P02768 | Serum albumin | 66.47/5.67 | 5 | 146 |
| Q62234 | Myomesin-1 | 185/5.8 | 5 | 207 |
| Q92679 | Myosin 7 | 223/5.6 | 2 | 120 |
| Q92679 | Myosin 7 | 223/5.6 | 6 | 207 |
| P68133 | Actin, alpha skeletal muscle | 41.82/5.23 | 18 | 1864 |
| P68032 | Actin alpha cardiac muscle 1 | 41.8/5.2 | 10 | 490 |
| O95831 | Apoptosis-inducing factor 1 | 55.8/6.9 | 6 | 268 |
| P68133 | Actin, alpha skeletal muscle | 41.82/5.23 | 8 | 245 |
| P68133 | Actin, alpha skeletal muscle | 41.82/5.23 | 8 | 372 |
| P01009 | Alpha-1-antitrypsin | 44.3/5.4 | 1 | 99 |
| P08670 | Vimentin | 53/5.0 | 18 | 683 |
| P04264 | Keratin, typ II cytoskeletal | 66/8.1 | 1 | 39 |
| Q02388 | Collagen alpha-1 chain | 293/5.9 | 1 | 19 |
| P10809 | 60 kDa heat shock protein | 61/5.7 | 13 | 693 |
| P36871 | Phosphoglucomutase 1 | 61.7/6.3 | 19 | 797 |
| Q9BWB5 | Pyruvate kinase isozymes M1/M2 | 58.5/8 | 10 | 421 |
| Q9BWB5 | Pyruvate kinase isozymes M1/M2 | 58.5/8 | 11 | 640 |
| Q9BWB5 | Pyruvate kinase isozymes M1/M2 | 58.5/8 | 10 | 433 |
| Q9BWB5 | Pyruvate kinase isozymes M1/M2 | 58.5/8 | 9 | 350 |
| P36871 | Phosphoglucomutase-1 | 61.7/6.3 | 1 | 60 |
| Q9BWB5 | Pyruvate kinase isozymes M1/M2 | 58.5/8 | 8 | 324 |
| P68133 | Actin, alpha skeletal muscle | 41.82/5.23 | 8 | 373 |
| P13645 | Creatine kinase M-type | 43/6.8 | 2 | 142 |
| P40123 | Adenylyl cyclase-associated protein2 | 53/5.9 | 4 | 135 |
| Q9BWB5 | Pyruvate kinase isozymes M1/M2 | 58.5/8 | 14 | 472 |
| P62736 | Actin aortic smooth muscle | 42.4/5.23 | 10 | 318 |
| Q8TD99 | Desmin | 53.6/5.2 | 22 | 861 |
| Q9BWB5 | Pyruvate kinase isozymes M1/M2 | 58.5/8 | 14 | 569 |
| Q9UBF9 | Myotilin | 55.4/9.2 | 22 | 867 |
| P02675 | Fibrinogen beta chain | 56.6/8.5 | 5 | 276 |
| P62736 | Actin aortic smooth muscle | 42.4/5.23 | 7 | 160 |
| Q9UBF9 | Myotilin | 55.4/9.2 | 13 | 478 |
| P06733 | Alpha enolase | 47/7 | 5 | 381 |
| P04264 | Keratin, typ II cytoskeletal | 66/8.1 | 3 | 122 |
| Q9UBF9 | Myotilin | 55.4/9.2 | 11 | 388 |
| Q8TD99 | Desmin | 53.6/5.2 | 23 | 1014 |
| Q9H0J4 | Glutamine-rich protein 2 | 180/6.2 | 1 | 37 |
| P06576 | ATP synthase subunit beta | 51.8/5 | 7 | 405 |
| Q9BWB5 | Pyruvate kinase isozymes M1/M2 | 58.5/8 | 15 | 735 |
| P07437 | Tubulin beta chain | 50/4.8 | 16 | 787 |
| Q8TD99 | Desmin | 53.6/5.2 | 39 | 1059 |
| Q8TD99 | Desmin | 53.6/5.2 | 24 | 588 |
| P25705 | ATP synthase subunit alpha | 55/8.3 | 2 | 145 |
| Q8TD99 | Desmin | 53.6/5.2 | 32 | 1584 |
| P25705 | ATP synthase subunit alpha | 55/8.3 | 24 | 846 |
| P13645 | Creatine kinase M-type | 43/6.8 | 8 | 393 |
| P06576 | ATP synthase subunit beta | 51.8/5 | 17 | 1410 |
| P06576 | ATP synthase subunit beta | 51.8/5 | 25 | 2255 |
| P25705 | ATP synthase subunit alpha | 55/8.3 | 9 | 436 |
| Q8TD99 | Desmin | 53.6/5.2 | 26 | 934 |
| Q16851 | UTP-glucose-1-phosphate uridylyltransferase | 56.8/8.1 | 18 | 831 |
| Q6NZI2 | Polymerase I and transcript release factor | 43/5.5 | 1 | 100 |
| P25705 | ATP synthase subunit alpha | 55/8.3 | 1 | 99 |
| Q02252 | Methylmalonate-semialdehyde dehydrogenase | 58/8.7 | 7 | 274 |
| P25705 | ATP synthase subunit alpha | 55/8.3 | 4 | 129 |
| P25705 | ATP synthase subunit alpha | 55/8.3 | 14 | 575 |
| P25705 | ATP synthase subunit alpha | 55/8.3 | 7 | 278 |
| P25705 | ATP synthase subunit alpha | 55/8.3 | 23 | 839 |
| P13929 | Beta-enolase | 46.8/7.7 | 6 | 241 |
| P68133 | Actin, alpha skeletal muscle | 41.82/5.23 | 5 | 276 |
| P13645 | Creatine kinase M-type | 43/6.8 | 10 | 450 |
| P07951 | Tropomyosin beta chain | 32.9/4.7 | 6 | 188 |
| P04264 | Keratin, typ II cytoskeletal | 66/8.1 | 2 | 80 |
| P35998 | 26S protease regulatory subunit 7 | 49/5.7 | 7 | 319 |
| P68133 | Actin, alpha skeletal muscle | 41.82/5.23 | 21 | 1184 |
| Q05639 | Elongation factor 1-alpha 2 | 50.5/9.1 | 9 | 204 |
| P06733 | Alpha enolase | 47/7 | 17 | 886 |
| Q9UKX2 | Myosin-2 | 224/5.6 | 23 | 638 |
| P12882 | Myosin-1 | 224/5.6 | 10 | 341 |
| P13929 | Beta-enolase | 46.8/7.7 | 23 | 867 |
| P13929 | Beta-enolase | 46.8/7.7 | 16 | 1010 |
| P68133 | Actin, alpha skeletal muscle | 41.82/5.23 | 9 | 438 |
| P13929 | Beta-enolase | 46.8/7.7 | 6 | 190 |
| P68133 | Actin, alpha skeletal muscle | 41.82/5.23 | 22 | 873 |
| P68133 | Actin, alpha skeletal muscle | 41.82/5.23 | 17 | 953 |
| P68133 | Actin, alpha skeletal muscle | 41.82/5.23 | 20 | 903 |
| P68133 | Actin, alpha skeletal muscle | 41.82/5.23 | 14 | 772 |
| P68133 | Actin, alpha skeletal muscle | 41.82/5.23 | 9 | 389 |
| P00558 | Phosphoglycerate kinase 1 | 44.5/8.3 | 19 | 706 |
| P48735 | Isocitrate dehydrogenase [NADP] | 51.3/8.9 | 17 | 525 |
| P12277 | Creatine kinase B-type | 42.9/5.3 | 5 | 199 |
| P22695 | Cytochrome b-c1 complex subunit 2 | 48.6/8.7 | 14 | 559 |
| P13645 | Creatine kinase M-type | 43/6.8 | 2 | 181 |
| P13645 | Creatine kinase M-type | 43/6.8 | 23 | 3575 |
| P62736 | Actin aortic smooth muscle | 42.4/5.23 | 7 | 399 |
| P00558 | Phosphoglycerate kinase 1 | 44.5/8.3 | 14 | 535 |
| P68133 | Actin, alpha skeletal muscle | 41.82/5.23 | 8 | 288 |
| P24752 | Acetyl-CoA acetyltransferase | 41.4/8.2 | 14 | 692 |
| P00505 | Aspartate aminotransferase | 44.7/8.9 | 6 | 318 |
| P82650 | 28S ribosomal protein S22 | 41/7.7 | 4 | 148 |
| P04075 | Fructose-bisphosphate aldolase A | 39.4/8.3 | 20 | 3954 |
| P04264 | Keratin, typ II cytoskeletal | 66/8.1 | 13 | 438 |
| P04406 | Glyceraldehyde-3-phosphate dehydrogenase | 35.9/8.6 | 13 | 242 |
| P04075 | Fructose-bisphosphate aldolase A | 39.4/8.3 | 13 | 452 |
| P04075 | Fructose-bisphosphate aldolase A | 39.4/8.3 | 13 | 452 |
| Q53S27 | Uncharacterized protein C2orf53 | 44.7/10.4 | 1 | 36 |
| P04075 | Fructose-bisphosphate aldolase A | 39.4/8.3 | 8 | 294 |
| P04406 | Glyceraldehyde-3-phosphate dehydrogenase | 35.9/8.6 | 4 | 96 |
| P40925 | Malate dehydrogenase | 36.3/6.9 | 11 | 324 |
| P04075 | Fructose-bisphosphate aldolase A | 39.4/8.3 | 6 | 337 |
| P04406 | Glyceraldehyde-3-phosphate dehydrogenase | 35.9/8.6 | 9 | 370 |
| P04406 | Glyceraldehyde-3-phosphate dehydrogenase | 35.9/8.6 | 2 | 124 |
| P04406 | Glyceraldehyde-3-phosphate dehydrogenase | 35.9/8.6 | 2 | 52 |
| P04406 | Glyceraldehyde-3-phosphate dehydrogenase | 35.9/8.6 | 14 | 2062 |
| P31415 | Calsequestrin-1 | 41.7/3.9 | 5 | 164 |
| 00757 | Fructose-1,6-bisphosphotase isozyme2 | 36.7/6.8 | 7 | 324 |
| P13805 | Troponin T,slow skeletal muscle | 32.8/5.9 | 2 | 91 |
| Q99460 | 26S proteasome non-ATPase regulatory subunit1 | 105.8/5.2 | 1 | 43 |
| P62736 | Actin aortic smooth muscle | 42.4/5.23 | 5 | 147 |
| O75112 | LIM domain-binding protein 3 | 77/8.5 | 7 | 3497 |
| P16152 | Carbonyl reductase [NADPH]1 | 30.2/8.5 | 6 | 445 |
| P21796 | Voltage-dependent anion-selective channel protein | 30.6/8.6 | 11 | 957 |
| Q99460 | 26S proteasome non-ATPase regulatory subunit1 | 105.8/5.2 | 1 | 34 |
| P07451 | Carbonic anhydrase 3 | 29.4/6.9 | 7 | 162 |
